# The low-density lipoprotein receptor contributes to carotenoid homeostasis by regulating tissue uptake and fecal elimination

**DOI:** 10.1016/j.molmet.2024.102007

**Published:** 2024-08-10

**Authors:** Anthony P. Miller, Walter C. Monroy, Gema Soria, Jaume Amengual

**Affiliations:** 1 Department of Food Science and Human Nutrition, University of Illinois Urbana Champaign, Urbana, IL, USA; 2 Division of Nutritional Sciences, University of Illinois Urbana Champaign, Urbana, IL, USA

**Keywords:** Enterocyte, Basolateral membrane, Retinoids

## Abstract

**Objective:**

Carotenoids are lipophilic plant molecules with antioxidant properties. Some carotenoids such as β-carotene also serve as vitamin A precursors, playing a key role in human health. Carotenoids are transported in lipoproteins with other lipids such as cholesterol, however, the mechanisms responsible for carotenoid storage in tissues and their non-enzymatic elimination remain relatively unexplored. The goal of this study was to examine the contribution of the low-density lipoprotein receptor (LDLR) in the bodily distribution and disposal of carotenoids.

**Methods:**

We employed mice lacking one or both carotenoid-cleaving enzymes as suitable models for carotenoid accumulation. We examined the contribution of LDLR in carotenoid distribution by crossbreeding these mice with Ldlr-/- mice or overexpressing LDLR in the liver.

**Results:**

Our results show that LDLR plays a dual role in carotenoid homeostasis by simultaneously favoring carotenoid storage in the liver and adipose tissue while facilitating their fecal elimination.

**Conclusions:**

Our results highlight a novel role of the LDLR in carotenoid homeostasis, and unveil a previously unrecognized disposal pathway for these important bioactive molecules.

## Highlights

• LDLR participates in carotenoid uptake in the liver and adipose tissue, the two main carotenoid reservoirs.
• We describe a non-enzymatic pathway for carotenoid fecal elimination.
• LDLR is partially responsible for fecal carotenoid elimination in mice.

## 1 Introduction

Carotenoids are a group of over 1,000 lipids responsible for most yellow to red coloration found in fruits and vegetables [1]. The consumption of carotenoid-rich foods, as well as high plasma carotenoid levels, correlates with positive health outcomes such as a reduced incidence of cardiometabolic diseases, cancer, and cognitive disorders [[2], [3], [4], [5], [6]]. As intact molecules, carotenoids mitigate oxidative stress by serving as potent antioxidants in lipid-rich environments. Examples of this are lutein and zeaxanthin in the eye, and skin carotenoids serving as photoprotective agents [7,8]. Through their cleavage products, provitamin A carotenoids contribute to sustain vision and regulate gene expression by supplying retinal and retinoic acid to cells, respectively [9].

Mammals express two carotenoid-cleaving enzymes: β-carotene oxygenase 1 (BCO1) and the BCO2. BCO1 is a cytosolic enzyme that primarily cleaves β-carotene to produce vitamin A, while BCO2 is a mitochondrial enzyme involved in the cleavage of a great variety of oxidized carotenoids such as lutein and zeaxanthin [[10], [11], [12]]. While humans accumulate substantial amounts of carotenoids in plasma and tissues, most experimental models in biomedical research, including mice, fail to mimic this phenotype. Hence, wild-type mice are not an adequate model to study the mechanisms regulating carotenoid distribution to tissues (reviewed in [13]). We overcame this limitation with the development and characterization of the *Bco1*^*−/−*^ and *Bco2*^*−/−*^ mice, which accumulate β-carotene and xanthophylls such as lutein, respectively [12,14].

Together with other lipids, carotenoids are transported in plasma bound to chylomicrons, very low and low-density lipoproteins [(V)LDL], and high-density lipoproteins (HDL). Additionally, several lipid-binding proteins and transporters have been reported to interact with carotenoids, suggesting common delivery pathways for these lipids [[15], [16], [17]]. For example, Tontonoz's group described the role Aster proteins play in the transport of cholesterol [18], which served as a steppingstone to identify AsterB as an intracellular carotenoid binding protein [15]. Also, membrane transporters such as ATP Binding Cassette Subfamily A Member 1 (ABCA1) and the scavenger receptor class B type I (SR-BI) participate in the transport of cholesterol and carotenoids across plasma membranes [16,[19], [20], [21], [22], [23], [24], [25]].

In 2016, Quadro's group described the role of the LDL receptor (LDLR) in hepatic β-carotene uptake by comparing *Ldlr*^*−/−*^ to *Ldlr*^*+/−*^ pregnant dams injected intraperitoneally with β-carotene [26]. However, no further studies explored whether LDLR influences the uptake of carotenoids other than β-carotene or whether LDLR participates in carotenoid homeostasis when these compounds are provided in the diet. In the present study, we aimed to establish the role of LDLR in carotenoid homeostasis. We show that LDLR contributes to carotenoid uptake in the liver and adipose tissue, which account for the two largest carotenoid reservoirs in the body [[27], [28], [29]]. We further show that LDLR participates in fecal β-carotene elimination, a novel pathway of carotenoid disposal.

## 2 Experimental procedures

### 2.1 Animals and diets

All studies were performed following the guidelines published in the NIH Guide for the Care and Use of Laboratory Animals [30]. The Institutional Animal Care and Use Committee of the University of Illinois at Urbana Champaign reviewed and approved the animal protocol. *Bco1*^−/−14^, *Bco2*^−/−12^, and *Bco1*^−/−^*Ldlr*^−/−31^ mice were used for the experiments described. All mice were backcrossed for eleven generations with C57BL/6 mice (Jax #000664) to generate congenic mice, as done in the past [32,41]. Mice were maintained at 24 °C in a 12 h/12 h light/dark cycle with *ad libitum* access to food and water. All mice were fed a non-purified breeder diet containing 15 IU vitamin A/g diet until reaching four weeks of age (Teklad global 18% protein diet, Envigo, Indianapolis, IN, US).

For all experiments, we matched comparable numbers of male and female mice for the different experimental groups. Typically, our experiments started when mice turned four weeks old, which is the time we selected to switch them to our experimental diets. For our studies, we utilized two types of diet: Standard and Western diets. Energy sources for Standard diets consisted of 16% fat, 64% carbohydrate, and 20% protein-derived calories, while % of energy in Western diets was divided into 41% fat, 39% carbohydrate, and 20% protein. Only Western diets contain 3% cholesterol.

Unless otherwise stated, diets were deprived of vitamin A to facilitate carotenoid uptake [33,34]. Diets containing vitamin A were enriched with 4 IU/g of vitamin A, as recommended by the former American Institute of Nutrition [35]. We incorporated carotenoids into the diets using water-soluble beadlets containing a proprietary formulation with DL-α-tocopherol, ascobyl palmitate, corn oil, fish gelatin, sucrose, and corn starch (DSM Ltd., Sisseln, Switzerland). Both β-carotene and lutein diets contained a final concentration of 50 mg carotenoid/kg diet. Diets without carotenoids contained placebo beadlets lacking carotenoids. Diets were prepared by Research Diets, Inc. (New Brunswick, NJ, US) by cold extrusion to protect carotenoids from heat. The concentration of carotenoids in the diet was consistent with our previous studies [12,31,32,[36], [37], [38]]. Table 1 contains a detailed description of all experimental diets.

**Table 1:** Composition of the diets utilized in this study.^a^

	SD + 50 mg BC/kg	WD + 50 mg BC/kg	SD no BC	WD no BC	SD + 50 mg Lut/kg	SD VAS	SD VAD
Protein (kcal)	20	20	20	20	20	20	20
Carbohydrate (kcal)	64	39	64	39	64	64	64
Fat (kcal)	16	41	16	41	16	16	16
Total	100	100	100	100	100	100	100
**Ingredient**	**g/kg diet**	**g/kg diet**	**g/kg diet**	**g/kg diet**	**g/kg diet**	**g/kg diet**	**g/kg diet**
Sucrose	100	212	100	212	100	100	100
Casein	200	200	200	200	200	200	200
Corn starch	397.5	72.8	397.5	72.8	397.5	397.5	397.5
Maltodextrin	132	100	132	100	132	132	132
Soybean oil	70	25	70	25	70	70	70
Lard	0	160	0	160	0	0	0
Cholesterol	0	3.08	0	3.08	0	0	0
Alphacel (cellulose)	50	50	50	50	50	50	50
Mineral mix (AIN93M-MX)	35	10	35	10	35	35	35
Vitamin mix (AIN93-VX)	10	10	10	10	10	10	10
l-Cysteine	1.8	3	1.8	3	1.8	1.8	1.8
Choline bitartrate	3	3	3	3	3	3	3
t-Butylhydroquinone	0.014	0	0.014	0	0.014	0.014	0.014
Lutein beadlets (5%)	0	0	0	0	1	0	0
β-Carotene Beadlets (10%)	0.5	0.5	0	0	0	0	0
Placebo Beadlets	0	0	1	1	0	1	1
Vitamin acetate (500 IU/kg)	0	0	0	0	0	0.007	0

a Abbreviations: SD, standard diet; BC, β-carotene; WD, Western diet; Lut, lutein; VAS, vitamin A sufficient; VAD, vitamin A deficient; IU, international units.

### 2.2 Tissue harvesting

In all experiments, we fasted the mice for up to 4 h before tissue collection. Mice were anesthetized by intraperitoneal injection of 80 mg ketamine and 8 mg xylazine/kg body weight followed by blood collection directly from the heart using EDTA-coated syringes. Mice were then perfused with a saline solution (0.9% NaCl in water) before collecting the liver, adipose tissues (gonadal and inguinal), and eyes. The small intestine was collected, and the attached adipose tissue was removed. We flushed the lumen of the intestine with 1× PBS to eliminate fecal matter, and then cut the organ into three pieces (duodenum, jejunum, and ileum). All organs were snap-frozen in liquid nitrogen and subsequently stored at −80 °C. Blood plasma was collected by centrifugation at 2000 × *g* for 10 min at 4 °C and immediately stored at −80 °C.

### 2.3 Intraperitoneal β-carotene administration

β-Carotene was administered to male and female *Bco1*^−/−^ and *Bco1*^−/−^*Ldlr*^−/−^ mice fed a carotenoid-free diet by intraperitoneal injection. A β-carotene emulsion was prepared as described previously [26]. Briefly, β-carotene (Thermo Fisher Scientific, Waltham, MA, USA) was combined with a mixture of ethanol, crempohor (Sigma, St. Louis, MO, USA), and 1× PBS (1:11:18 ratio) to create a final concentration of approximately 3.5 g β-carotene/L solution. Mice were sedated initially with 4–5% isoflurane and maintained with 1–2% isoflurane. Mice were then injected with a dose of 35 μg β-carotene/g body weight.

### 2.4 Liver-specific adeno-associated viral (AAV) constructs and injections

All AAVs were generated by the University of Pennsylvania Vector Core facility using an AAV8.TBG.PI.eGFP.WPRE.bGH as a backbone (AAV-GFP, Addgene plasmid #105535 originally generated by James Wilson http://n2t.net/addgene:105535). To target hepatocytes, all AAVs included the hepatocyte-specific thyroxine-binding globulin (TBG) promoter. We injected the mice retro-orbitally with 1 × 10^11^ viral genomic copies/mouse under anesthesia (isoflurane).

### 2.5 Collection of feces

To analyze carotenoid excretion, we collected feces every 12 h from individually housed animals during the four days of carotenoid-free diet feeding. As previously described, we placed grates at the bottom of the cage to limit coprophagy [31,39].

### 2.6 Plasma lipoprotein separation

Lipoproteins were fractionated using a Shimadzu HPLC system incorporating two consecutive Superose 6 10/300 GL columns (GE Healthcare, Boston, MA) running on a Shimadzu HPLC system (Columbia, MD) in an aqueous solution. Eluted samples were collected and stored at −20 °C. Lipoprotein species were identified by quantifying cholesterol and triglyceride content in each fraction and by probing for apolipoprotein B (apoB) and apolipoprotein A-I (apoA-I) levels, as described in the past [39]. We also separated HDL from non-HDL by using the HDL cholesterol assay kit (FUJIFILM Wako Diagnostics, Mountain View, CA).

Total cholesterol and triglyceride measurements were performed using standard colorimetric assays (FUJIFILM Wako Diagnostics) from 5 μL of plasma or 100 μL of FPLC fraction.

### 2.7 Gallbladder isolation and bile acid measurements

For bile acid quantifications, whole gallbladders were collected and placed in 1.5 mL tubes. The gallbladders were punctured with a needle to release their contents. Samples were centrifuged at room temperature at 1000 × *g*, and the supernatant was collected to measure bile acid content. All bile samples were diluted in sterilized 0.9% saline (1:20). Carotenoids in the bile were normalized to total bile acid content in the sample, which was estimated using the Total Bile Acids kit (#DZ042A-K; Diazyme, Poway, CA).

### 2.8 HPLC analysis of carotenoids, retinyl esters, and free retinol

Carotenoids and retinoids were extracted from 70 μL of plasma, 500 μL pooled FPLC fractions, one whole eyecup, 30 mg feces, or tissue homogenates in 1× PBS containing 10 mg of tissue. Plasma, liver, and eye homogenates were added to a mixture of 200 μL of ethanol and 400 μL acetone (organic phase). Carotenoids and retinoids were extracted by adding 500 μL of hexane (inorganic phase). We centrifuged the mixture at 500 × *g* for 1 min and transferred the upper layer (hexane) to a 1.5 mL Eppendorf tube.

Feces and adipose tissues (iWAT and gWAT) were saponified before carotenoid and retinoid extractions. First, feces were dried using a SpeedVac vacuum concentrator (Thermo Fisher Scientific Inc., Waltham, MA) and ground into powder. For all samples, we saponified approximately 30–50 mg of material with 200 μL of ethanol, 100 μL of 12% w/v pyrogallol (Sigma) in ethanol, and 200 μL of 30% w/v KOH at 37 °C for 2 h. Total lipid content was extracted twice with a mixture of diethyl ether:hexane:ethanol (66:33:1). If water was present in the sample, we performed a second extraction following the same method used for plasma, liver, and eyes. We dried supernatants using a Speed Vac for 25 min and reconstituted them with 200 μL of mobile phase hexane:ethyl acetate (80:20).

All extractions were conducted under a dim yellow safety light, as previously described [40]. For molar quantifications of carotenoids and retinoids, the HPLC was scaled with a standard curve using parent compounds, and extracts were separated in a Zorbax Sil column (Agilent Technologies, Santa Clara, CA) with an 80% hexane and 20% ethyl acetate (v:v) mobile phase. Carotenoids and retinoids were identified using commercially available standards and comparing elution times and spectra to the samples.

### 2.9 Immunoblotting

For the determination of hepatic levels of GFP and LDLR, proteins were extracted from liver lysates in RIPA buffer (50 mM Tris pH 7.4, 150 mM NaCl, 0.25% sodium deoxycholate, 1% Nonidet P-40). Total protein amounts were quantified using the Pierce® BCA Protein Assay Kit (Thermo). ApoB and apoA-I were quantified in 10 μl of FPLC fractions or 20–80 μg of protein homogenate. All samples were separated by SDS-PAGE and subsequently electroblotted onto 0.45 μm pore size nitrocellulose membranes (apoB) or 0.22 μm PVDF (apoA-I, GFP, LDLR) (Bio-Rad, Hercules, CA). Membranes were blocked with fat-free milk powder (5% w/v) dissolved in Tris-buffered saline (15 mM NaCl and 10 mM Tris/HCl, pH 7.5) containing 0.01% Tween 100 (TBS-T), washed, and incubated overnight at 4 °C with either goat anti-apoB (Millipore-Sigma, Burlington, MA), goat anti-apoA-I (Meridian Life Science Inc, Memphis, TN), mouse anti-GFP (Santa Cruz Biotechnologies, Dallas, TX), mouse anti-LDLR (Santa Cruz Biotechnologies), or mouse anti-β-actin (Sigma) as a housekeeping control. Bands were typically detected using infrared fluorescent-labeled secondary antibodies prepared at 1:15,000 dilution in TBS-T with 5% fat-free milk powder after incubation for 1 h at room temperature.

### 2.10 mRNA isolation and PCR analysis

Total RNA was isolated with Trizol reagent (Thermo) according to the manufacturer's instructions. RNA purity and concentration were measured with a Nanodrop spectrophotometer (Thermo). One μg of total RNA was reverse transcribed to cDNA with the Applied Biosystems retro-transcription kit (Applied Biosystems, Carlsbad, CA). Quantitative real-time PCRs were performed using TaqMan Fast Advanced Master Mix (Applied Biosystems) or SYBR reagents (Applied Biosystems) and primers (Integrated DNA Technologies, Coralville, IA) or probes (Applied Biosystems) for the following genes: *Ldlr*: Mm00440169_m1, *Gfp*: Mr03989638_mr, intestine-specific homeobox (*Isx*, 5′-ATC TGG GCT TGT CCT TCT CC-3′ and 5′-TTT TCT CTT CTT GGG GCT GA-3′), scavenger receptor class B type 1 (*Scarb1/SR-BI*, Mm00450234_m1). Glyceraldehyde-3-phosphate dehydrogenase *(Gapdh,* 5′-TTGGCATTGTGGAAGGGCTCAT-3′ and 5′-GATGACCTTGCCCACAGCCTT-3′) was used as a housekeeping control. Gene expression analyses were performed with the StepOnePlus Real-Time PCR System (Applied Biosystems) and the ΔΔCt calculation method.

### 2.11 Statistical analyses

Data are expressed as means ± standard error of the mean (SEM). Statistical differences were analyzed using GraphPad Prism software (GraphPad Software Inc., San Diego, CA). The distribution normality of sample groups was analyzed using the D'Agostino-Pearson omnibus and the Shapiro–Wilk normality tests. Statistical differences were evaluated by two-tailed Student t-testing between groups of two, two-way ANOVA, or linear regression analysis. Statistical significance was set at p < 0.05.

## 3 Results

### 3.1 Whole-body knockout of LDLR reduces hepatic and adipose β-carotene levels

**Figure 1:**
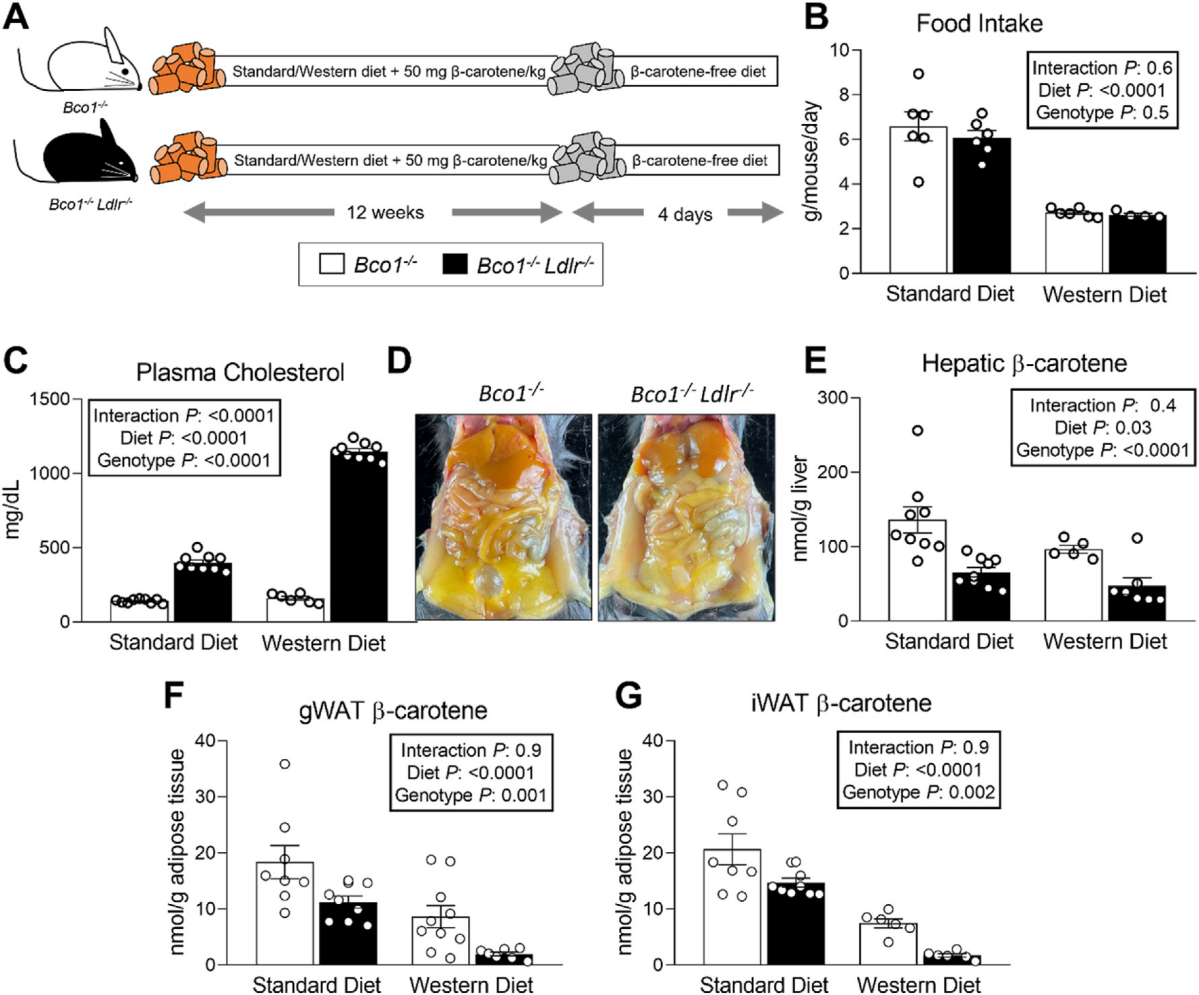
**Whole-body ablation of LDLR reduces hepatic and adipose β-carotene levels in mice. (A)** Experimental design. Four-week-old *Bco1*^*−/−*^ and *Bco1*^*−/−*^*/Ldlr*^*−/−*^ mice were fed a Standard or Western diet containing 50 mg/kg of β-carotene for 12 weeks and then switched to either Standard or Western β-carotene-free diet for four days before harvesting plasma and tissues for analysis. **(B)** Average food intake of *Bco1*^*−/−*^*and Bco1*^*−/−*^*/Ldlr*^*−/−*^ mice fed a Standard or Western diet. **(C)** Total plasma cholesterol levels in *Bco1*^*−/−*^ and *Bco1*^*−/−*^*Ldlr*^*−/−*^ mice at 12 weeks. **(D)** Representative image of carotenoid accumulation in tissues. **(E)** Hepatic, **(F)** gonadal white adipose tissue (gWAT), and **(G)** inguinal WAT (iWAT) β-carotene levels measured by HPLC. n = 6 to 9 mice/group. Statistical differences were evaluated by two-way ANOVA. Overall p-values are displayed in the box.

β-Carotene injected intraperitoneally is taken up by the liver via the LDLR [26]. Whether the uptake of dietary β-carotene follows the same pathway remains unanswered. To answer this question, we first compared the accumulation of β-carotene in age and sex-matched *Bco1*^*−/−*^ and *Bco1*^*−/−*^*Ldlr*^*−/−*^ mice. Because *Ldlr*^*−/−*^ mice tend to develop hypercholesterolemia [41], we performed parallel experiments in mice eating Standard and a Western-type diets containing 50 mg of β-carotene/kg diet (Standard-β-carotene and Western-β-carotene). Four days before tissue harvest, we switched the mice to a carotenoid-free diet (Figure 1A) to quantify intestinal β-carotene content and measure fecal β-carotene elimination. We did not observe differences in final body weight between genotypes or diet (data not shown). Mice eating Standard-β-carotene diet consumed more food per day than those eating Western-β-carotene diet, however we did not observe differences in food consumption between genotypes (Figure 1B). As expected [41], the absence of LDLR favored the accumulation of plasma cholesterol in mice, especially in those fed Western-β-carotene diet (Figure 1C).

Upon visual examination, *Bco1*^*−/−*^ mice accumulated more β-carotene in tissues in comparison to *Bco1*^*−/−*^*Ldlr*^*−/−*^ mice (Figure 1D). Indeed, hepatic β-carotene stores were depleted in mice lacking LDLR, independently of the diet (Figure 1E). Similarly, white adipose tissue (WAT) stores in two different depots were lower in *Bco1*^*−/−*^*Ldlr*^*−/−*^ mice than in *Bco1*^*−/−*^ mice (Figure 1F, G).

### 3.2 Circulating β-carotene increases in the absence of LDLR

**Figure 2:**
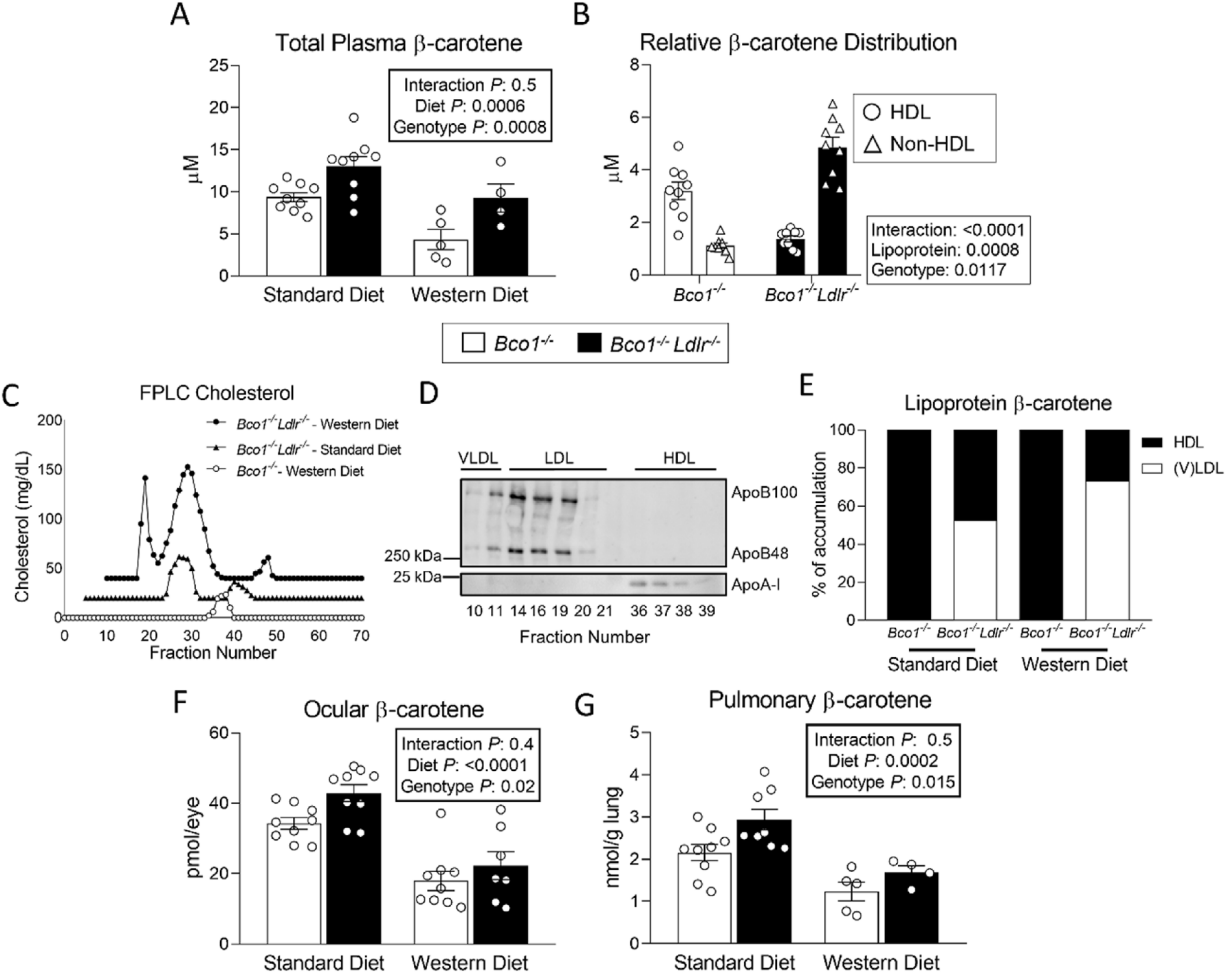
**Circulating β-carotene increases in the absence of LDLR.** Four-week-old *Bco1*^*−/−*^ and *Bco1*^*−/−*^*/Ldlr*^*−/−*^ mice were fed a Standard or Western diet containing 50 mg/kg of β-carotene for 12 weeks and then switched to either the Standard or Western β-carotene-free diet for four days before harvesting plasma and tissues for analysis. **(A)** Plasma β-carotene levels in *Bco1*^*−/−*^ and *Bco1*^*−/−*^*/Ldlr*^*−/−*^ mice. **(B)** Relative β-carotene distribution in HDL and non-HDL cholesterol. **(C)** Cholesterol distribution in FPLC-fractioned plasma (data pooled from three mice/group) and **(D)** western blot confirming the identity of the different lipoprotein species. **(E)** Accumulation (in %) of β-carotene in lipoprotein fractions**. (F)** Ocular and **(G)** pulmonary β-carotene levels measured by HPLC. N = 4 to 9 mice/group. Statistical differences were evaluated by two-way ANOVA. Overall p-values are displayed in the box.

We next sought to examine if the ablation of LDLR impacts plasma β-carotene concentration. *Bco1*^*−/−*^*Ldlr*^*−/−*^ mice displayed greater circulating β-carotene compared to *Bco1*^*−/−*^ mice fed either a Standard-β-carotene or Western-β-carotene diet. Mice fed Standard-β-carotene diet accumulated greater plasma β-carotene levels than those fed Western-β-carotene diet, independently of their genotype (Figure 2A).

We next sought to investigate the distribution of β-carotene among lipoprotein species. Under fasting conditions, carotenoids distribute between HDL and non-HDL particles in humans [42], while HDL is the main carrier of circulating carotenoids in *Bco1*^*−/−*^ and *Bco2*^*−/−*^ mice [43]. To examine the lipoprotein carotenoid composition of our phenotypes, we first precipitated plasma samples from mice fed a Standard-β-carotene diet using an HDL cholesterol assay kit (Wako). In *Bco1*^*−/−*^ mice, HDL was the main β-carotene transporter, while most β-carotene in *Bco1*^*−/−*^*Ldlr*^*−/−*^ mice was present in non-HDL lipoproteins (Figure 2B). To confirm these findings, we fractioned pooled plasma samples by FPLC (Figure 2C, D). *Bco1*^*−/−*^*Ldlr*^*−/−*^ mice accumulated (V)LDL species in their plasma, while we could only detect HDL lipoproteins in *Bco1*^*−/−*^ mice, irrespective of diet (Figure 2C). We collected the (V)LDL and HDL fractions and quantified β-carotene content by HPLC. The distribution of β-carotene in the HDL and (V)LDL fractions of *Bco1*^*−/−*^*Ldlr*^*−/−*^ mice fed Standard-β-carotene or Western-β-carotene diets followed a 47:53 and 27:73 ratio, respectively, while the totality of β-carotene in *Bco1*^*−/−*^ mice was in the HDL fraction (Figure 2E).

Ocular and pulmonary β-carotene levels followed a comparable pattern to the plasma, that is, mice fed Standard-β-carotene diet displayed greater β-carotene levels in tissues than those fed Western-β-carotene diet, and *Bco1*^*−/−*^*Ldlr*^*−/−*^ mice accumulated more β-carotene than *Bco1*^*−/−*^ mice (Figure 2F, G). As expected, our experimental conditions failed to result in vitamin A deficiency [31,37,44]. Importantly, the absence of LDLR did not affect vitamin A homeostasis under our experimental conditions (Supplementary Figure 1).

### 3.3 Hepatic LDLR overexpression promotes carotenoid uptake in the liver and depletes extrahepatic carotenoid pools

**Figure 3:**
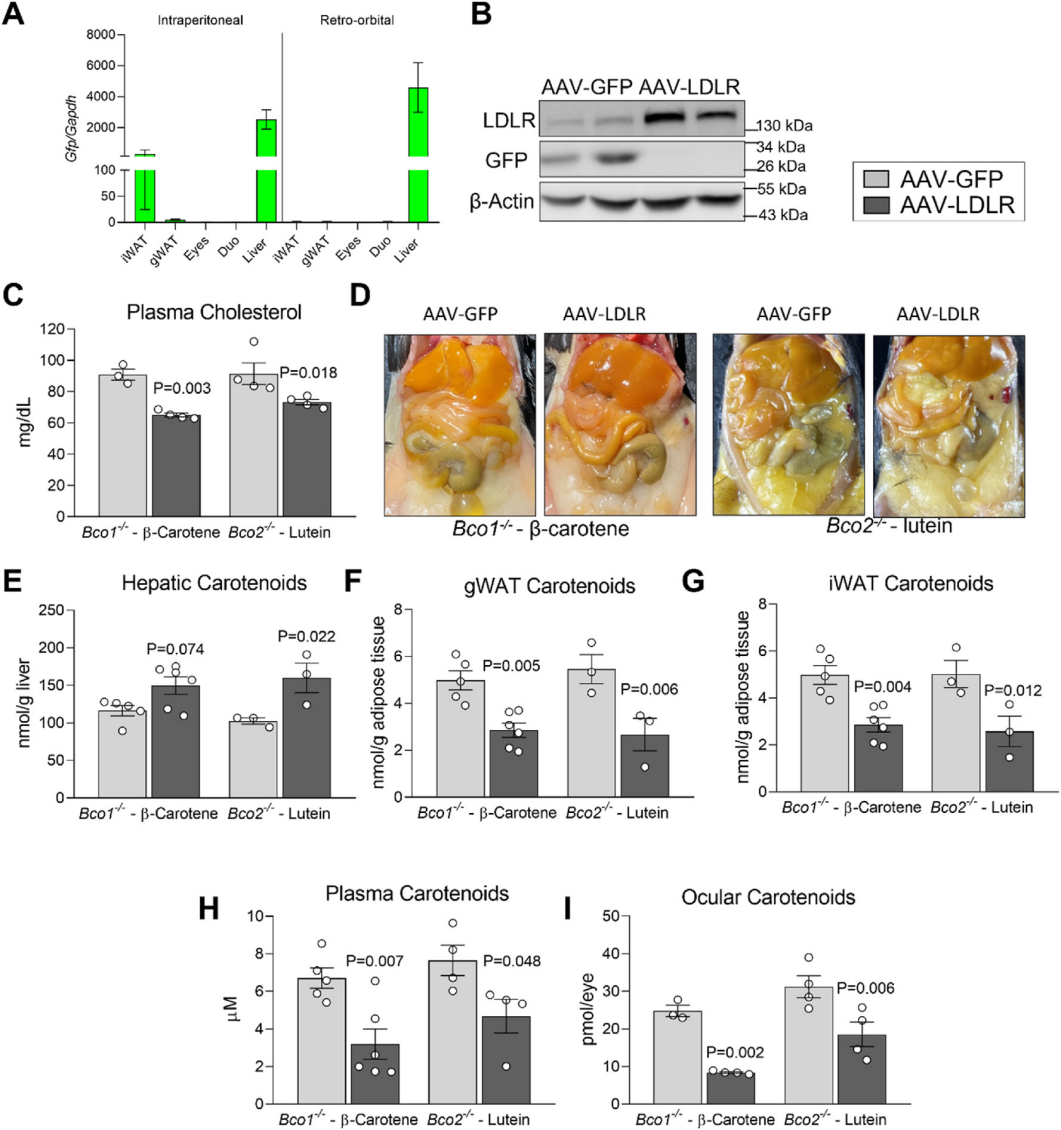
**Hepatic LDLR overexpression promotes carotenoid uptake in the liver and depletes extrahepatic carotenoid pools. (A)** mRNA levels of GFP in wild-type mice injected retro-orbitally or intraperitoneally with 1 × 10^11^ genomic copies/mouse. **(B)** Western blot analysis confirming the expression of GFP and LDLR in mice injected with AAV-GFP and AAV-LDLR, respectively. β-actin served as loading control. Each lane represents a different mouse. (**C–I**) *Bco1*^*−/−*^ and *Bco2*^*−/−*^ mice were injected retro-orbitally with 1 × 10^11^ genomic copies/mouse of AAV-GFP or AAV-LDLR. Mice were then fed a Standard diet supplemented with 50 mg/kg β-carotene (*Bco1*^*−/−*^ mice) or 50 mg/kg lutein (*Bco2*^*−/−*^ mice) for 4 weeks. **(C)** Total plasma cholesterol levels in *Bco1*^*−/−*^ and *Bco2*^*−/−*^ mice at 4 weeks. **(D)** Representative image of carotenoid accumulation in tissues. **(E)** Hepatic, **(F)** gonadal white adipose tissue (gWAT), **(G)** inguinal WAT (iWAT), **(H)** plasma, and **(I)** ocular carotenoid levels measured by HPLC. N = 3 to 6 mice/group. Statistical differences were evaluated by two-tailed Student's t-test. p-values are represented for each comparison.

Approximately 70% of circulating cholesterol is taken-up by hepatic LDLR [45]. Therefore, we aimed to explore whether hepatic LDLR expression modulates the uptake of β-carotene and lutein, a common xanthophyll in the human diet. To this end, we injected *Bco1*^*−/−*^ or *Bco2*^*−/−*^ mice with either AAV-LDLR or AAV-GFP (control) four weeks after birth. Preliminary studies showed that the retro-orbital injection route limited GFP expression to the liver to a greater degree than the intraperitoneal route, prompting us to inject the mice retro-orbitally (Figure 3A). After injections, we fed *Bco1*^*−/−*^ and *Bco2*^*−/−*^ mice with Standard-β-carotene or a Standard diet containing 50 mg of lutein/kg for four weeks, respectively. Western blot analyses confirmed the upregulation of either GFP or LDLR in total liver homogenates (Figure 3B), and plasma cholesterol levels confirmed the functionality of our AAV-LDLR (Figure 3C).

Upon tissue harvesting, we noted a visual difference in tissue carotenoid content between experimental groups that was especially obvious in the liver (Figure 3D). Indeed, hepatic β-carotene and lutein levels increased in those mice injected with AAV-LDLR in comparison to AAV-GFP-injected controls (Figure 3E). AAV-LDLR injections also depleted adipose, plasma, and ocular carotenoid levels for both cohorts in comparison to littermates injected with AAV-GFP (Figure 3F–I). We obtained comparable results in *Bco1*^*−/−*^*Ldlr*^*−/−*^ mice injected with AAV-LDLR or AAV-GFP and fed β-carotene for four weeks (Supplementary Figure 2).

### 3.4 Fecal carotenoid elimination depletes β-carotene stores

**Figure 4:**
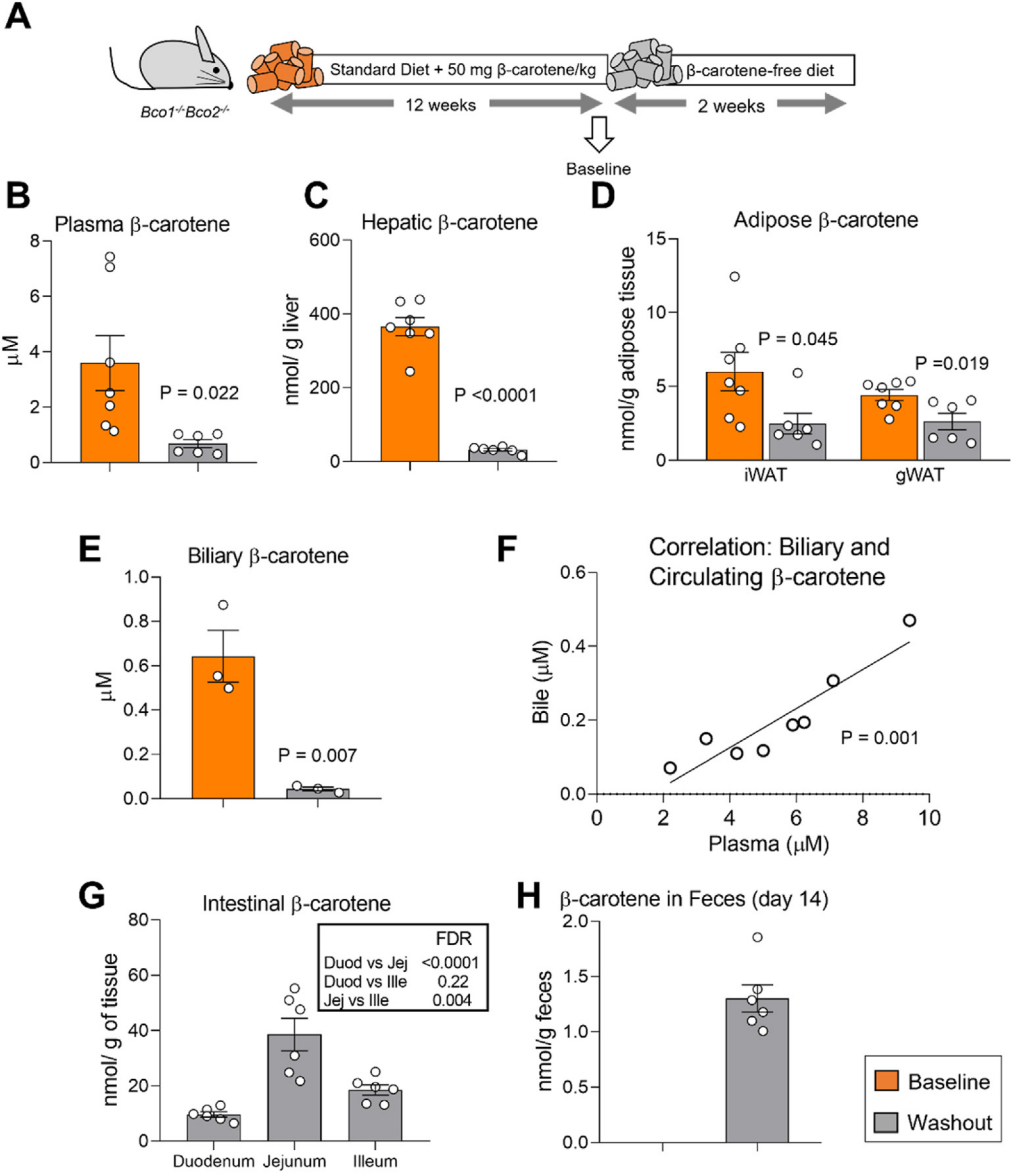
**Fecal carotenoid elimination depletes β-carotene stores over time. (A)** Experimental design. *Bco1*^*−/−*^*Bco2*^*−/−*^ mice were fed a Standard diet containing 50 mg/kg β-carotene for 12 weeks. One subset of mice was sacrificed and used as a Baseline group, and the rest were switched to a β-carotene-free diet for two weeks (Washout group). **(B)** Plasma, **(C)**hepatic, **(D)** inguinal white adipose tissue (iWAT) and gonadal WAT (gWAT), and **(E)** biliary β-carotene levels at the moment of the sacrifice. **(F)** Correlation between bile and plasma β-carotene concentrations in *Bco1*^*−/−*^ mice fed a Standard diet containing 50 mg/kg β-carotene for 2, 6, and 12 weeks. **(G)** Accumulation of β-carotene in the intestine, and **(H)** fecal β-carotene levels the Washout group after two weeks on the β-carotene-free diet. N = 3 to 7 mice/group. Statistical differences were evaluated by two-tailed Student's t-tests (p-values are represented for each comparison) and two-way ANOVA (p-values in the box).

To gain further insights into the impact of fecal carotenoid elimination on β-carotene homeostasis, we used *Bco1*^*−/−*^*Bco2*^*−/−*^ mice fed the Standard-β-carotene diet for 12 weeks, which accumulate β-carotene as *Bco1*^*−/−*^ mice do [46]. After 12 weeks, we sacrificed a subset of mice (Baseline) and switched the remaining mice to a carotenoid-free diet for two weeks before tissue harvesting (Washout, Figure 4A). HPLC analyses revealed a sharp reduction in circulating and hepatic β-carotene stores in the Washout group in comparison to Baseline mice (Figure 4B, C). WAT β-carotene stores followed the same pattern as the liver, but this reduction was less pronounced, indicating that the dynamics of β-carotene mobilization differs between the two major reservoirs (Figure 4D).

As reported by Leo and colleagues for human bile [47], we also detected the presence of β-carotene in the bile of *Bco1*^*−/−*^*Bco2*^*−/−*^ mice fed β-carotene. Biliary β-carotene was depleted in the Washout group in comparison to Baseline mice (Figure 4E). Because human bile content is directly associated to plasma levels [47], we next sacrificed a separate subset of *Bco1*^*−/−*^ mice fed the Standard-β-carotene diet and sacrificed them at different time points to collect the gallbladder and plasma. HPLC quantifications showed that circulating and biliary β-carotene correlated positively in *Bco1*^*−/−*^ mice (Figure 4F).

We also quantified intestinal β-carotene levels in the Washout group. We detected the presence of β-carotene in all tissue samples, showing that the jejunum is enriched in comparison to the duodenum and the ileum (Figure 4G). β-Carotene was also present in fecal samples collected the day of tissue harvesting in the washout group (Figure 4H). In a separate experiment, we detected β-carotene even after three weeks on a carotenoid-free diet (data not shown), suggesting that the fecal elimination of β-carotene probably occurs until the complete depletion of β-carotene stores.

### 3.5 LDLR participates in the intestinal elimination of β-carotene

The cholestane ring in the structure of cholesterol limits its enzymatic degradation in most living organisms [48]. To prevent the excessive accumulation of cholesterol, mammals eliminate this compound in their feces by modifying the cholestane ring to form bile acids, and by eliminating intact cholesterol via transintestinal cholesterol elimination (TICE). LDLR is a major contributor to fecal cholesterol elimination by participating in the uptake of circulating lipoproteins in hepatocytes and enterocytes to facilitate bile acids synthesis and TICE, respectively [49,50]. Therefore, we questioned whether LDLR participates in the fecal elimination of β-carotene.

**Figure 5:**
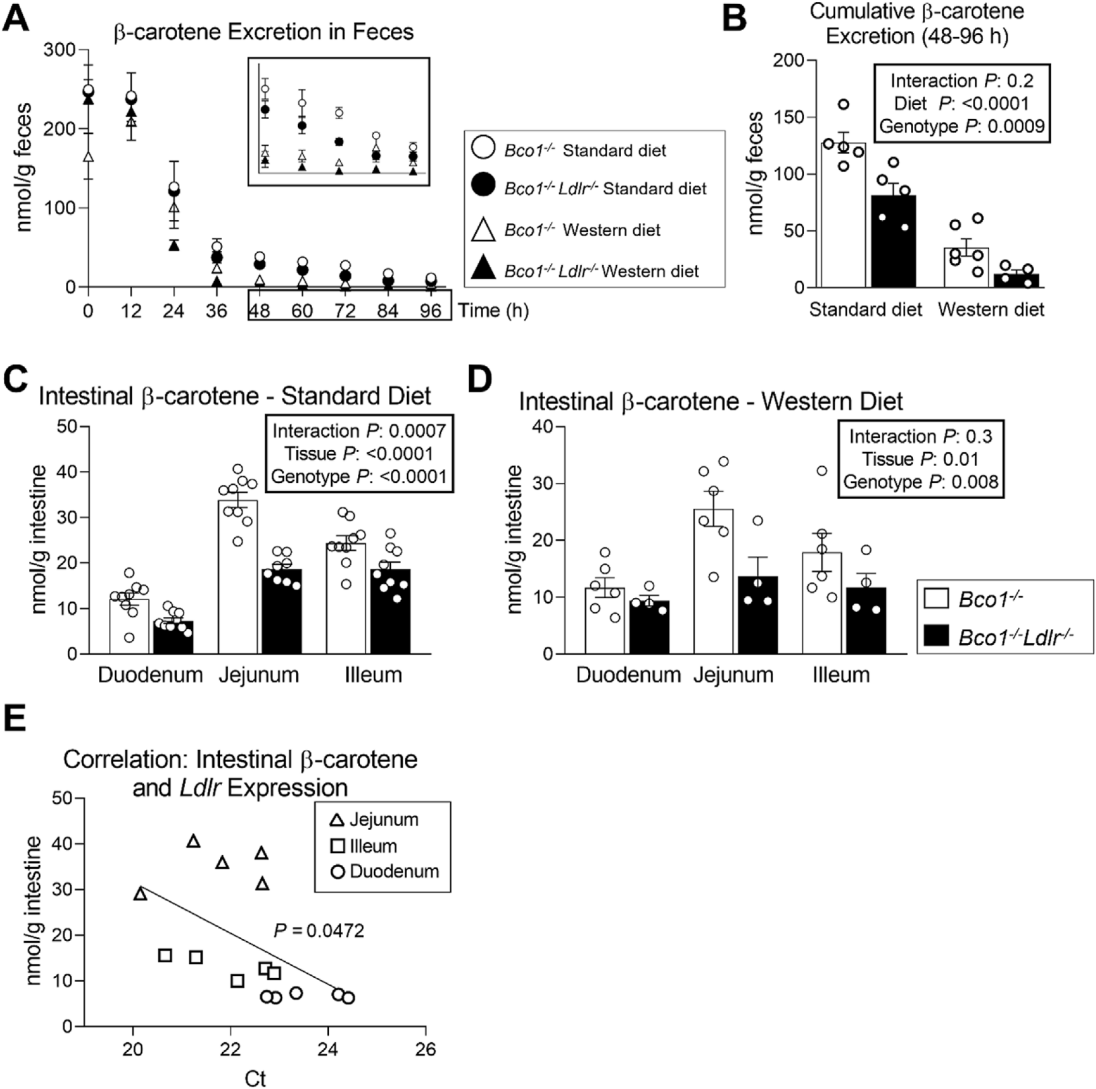
**LDLR contributes to fecal β-carotene elimination.** Four-week-old *Bco1*^*−/−*^ and *Bco1*^*−/−*^*/Ldlr*^*−/−*^ mice were fed a Standard or Western diet containing 50 mg/kg of β-carotene for 12 weeks and then switched to either a Standard or Western β-carotene-free diet for four days before harvesting plasma and tissues for analyses. We collected feces every 12 h to examine fecal β-carotene levels. **(A)** Fecal β-carotene levels in *Bco1*^*−/−*^*and Bco1*^*−/−*^*Ldlr*^*−/−*^ mice, and **(B)** cumulative β-carotene excretion over the final 48 h of feces collection. **(C)** β-carotene levels in the intestine of *Bco1*^*−/−*^*and Bco1*^*−/−*^*Ldlr*^*−/−*^ mice fed with a Standard diet, **(D)** or Western diet supplemented with β-carotene. **(E)** Correlation between intestinal β-carotene levels and LDLR expression. N = 4 to 9 mice/group. Statistical differences were evaluated by two-way ANOVA or linear correlation. Overall p-values are displayed in the box.

First, we compared the fecal elimination of β-carotene between *Bco1*^*−/−*^ and *Bco1*^*−/−*^*Ldlr*^*−/−*^ mice fed β-carotene for 12 weeks. To prevent contamination of ingested β-carotene in the feces, we switched all the mice to a carotenoid-free diet for four days before tissue harvest (Figure 1A). For both genotypes, we observed a sharp decline in fecal β-carotene content after 24 h, coinciding within the time required to empty the gastrointestinal tract in mice [51]. Mice continuously eliminated β-carotene in the feces until tissue harvesting, which occurred 96 h after diet switch (Figure 5A). To estimate the net elimination without the interference of residual dietary β-carotene, we limited the quantifications to fecal β-carotene collected between 48 h and 96 h after the dietary switch (Figure 5A, insert). HPLC quantifications revealed 1.5-fold greater β-carotene excretion in *Bco1*^*−/−*^ mice compared to *Bco1*^*−/−*^*Ldlr*^*−/−*^ mice fed a Standard-β-carotene diet, and 3-fold greater excretion from mice fed a Western-β-carotene (Figure 3B).

Next, we quantified β-carotene levels in the duodenum, jejunum, and ileum. Intestinal β-carotene was greater in *Bco1*^*−/−*^ mice in comparison to *Bco1*^*−/−*^*Ldlr*^*−/−*^ mice, regardless of the diet. In agreement with our previous experiment (Figure 4G), the jejunum was the section with the greatest β-carotene content in *Bco1*^*−/−*^ mice (Figure 5C, D). Figure 5E displays a correlation between *Ldlr* expression and β-carotene levels in different portions of the intestine from *Bco1*^*−/−*^ mice eating the Standard-β-carotene diet, suggesting that LDLR drives the reverse transport of plasma β-carotene to the feces.

**Figure 6:**
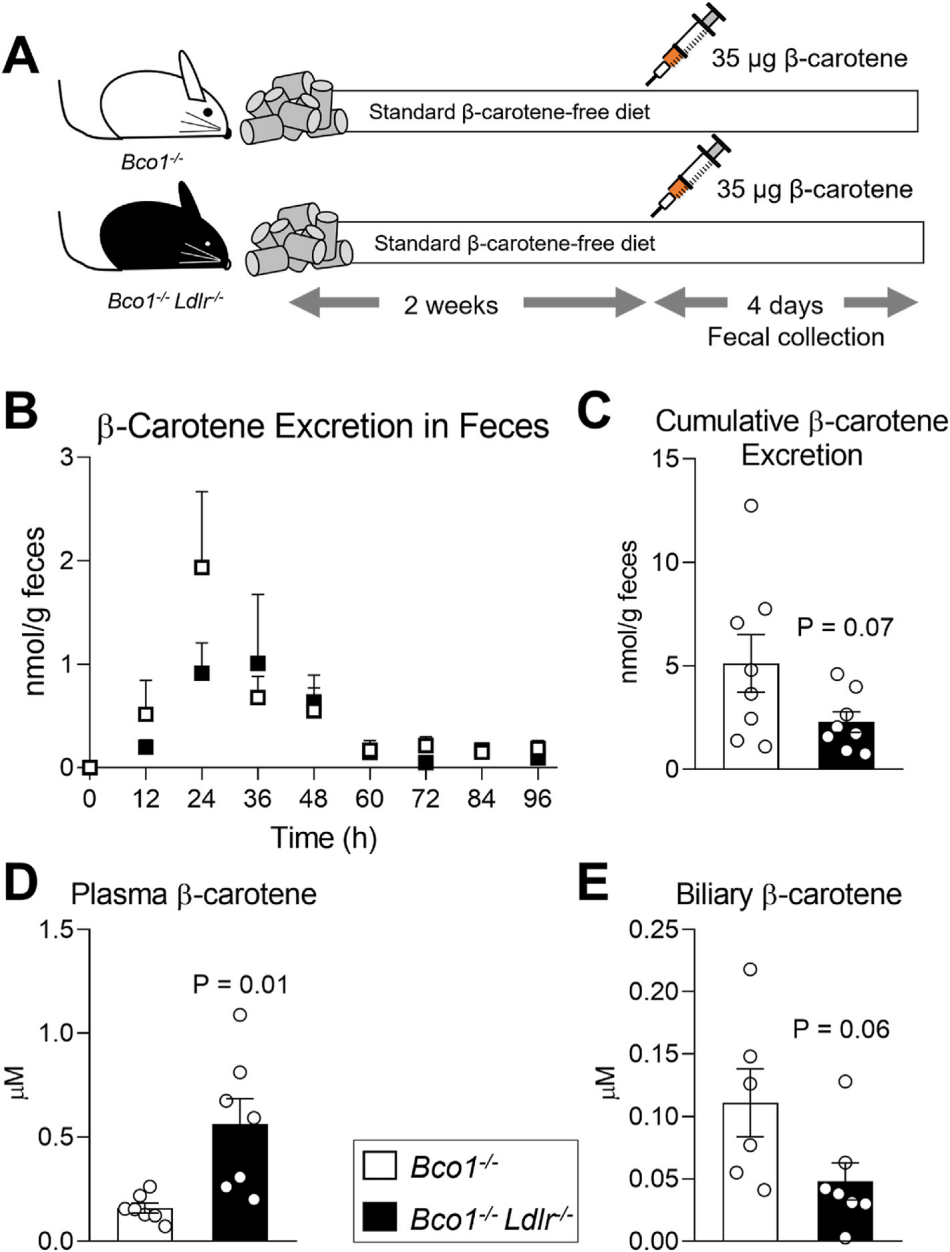
**β-Carotene administered intraperitoneally is eliminated in the feces. (A)** Experimental design. *Bco1*^*−/−*^ and *Bco1*^*−/−*^*Ldlr*^*−/−*^ mice fed a carotenoid-free diet were injected intraperitoneally with a single dose of 35 μg β-carotene/g body weight. **(B)** Fecal β-carotene levels during the 96 h after intraperitoneal injection measured by HPLC. **(C)** Cumulative β-carotene levels in feces over 96 h measured by HPLC. **(D)** Plasma and **(E)** biliary β-carotene levels. N = 4 to 9 mice/group. Statistical differences were evaluated by two-tailed Student's t-test. p-values are represented for each comparison.

Lastly, we aimed to confirm that systemic β-carotene can be disposed intact in the feces. We injected a single dose of β-carotene intraperitoneally into *Bco1*^*−/−*^ and *Bco1*^*−/−*^*Ldlr*^*−/−*^ mice fed a carotenoid-free diet for two weeks. Four days later, we sacrificed the mice for carotenoid analyses (Figure 6A). We observed a sharp increase in fecal β-carotene 24 h after injection for both genotypes (Figure 6B). Cumulative fecal β-carotene elimination followed an increasing trend in *Bco1*^*−/−*^ mice compared to *Bco1*^*−/−*^*Ldlr*^*−/−*^ mice (Figure 6C). Circulating β-carotene levels remained higher in *Bco1*^*−/−*^*Ldlr*^*−/−*^ mice in comparison to *Bco1*^*−/−*^ mice (Figure 6D), while the biliary content of β-carotene followed the opposite pattern but failed to reach statistical significance (Figure 6E).

### 3.6 SR-BI does not contribute to the intestinal elimination of β-carotene

**Figure 7:**
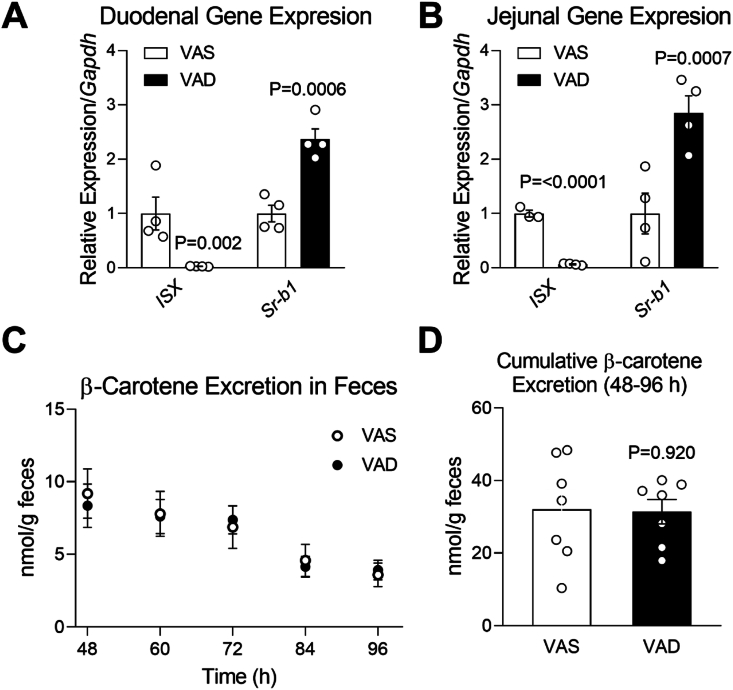
**Impact of vitamin A on the elimination of fecal β-carotene in *Bco1***^***−/−***^***/Ldlr***^***−/−***^**mice.***Bco1*^*−/−*^*/Ldlr*^*−/−*^ mice were fed a Standard diet supplemented with 50 mg/kg β-carotene for 4 weeks. Mice were then switched to either a vitamin A-sufficient (VAS) or vitamin A-deficient (VAD) diet without carotenoids for four days. **(A)** Duodenal and **(B)** Jejunal mRNA expression of intestine-specific homeobox (*ISX*) and scavenger receptor class B type 1 (*SR-BI*). *Gapdh* levels were used as a housekeeping control. **(C)** β-Carotene levels in feces between 48 and 96 h after the diet switch and **(D)** Cumulative β-carotene excretion levels. n = 3 to 7 mice/group. Statistical differences were evaluated by two-tailed Student's t-test. p-values are represented for each comparison.

The absence of LDLR does not completely prevent fecal β-carotene disposal, suggesting that other carotenoid transporters could contribute to this process. Among the potential candidates, we examined the contribution of intestinal SR-BI because (1) it has a well-documented role in the uptake of dietary carotenoids [33,[52], [53], [54]], (2) it is expressed in both the apical and the basolateral membrane of the enterocyte [55], (3) is the main receptor for HDL [56], a major carrier of β-carotene in our experimental conditions (Figure 2E), and (4) SR-BI expression levels in the enterocyte, but not other cell types, can be easily manipulated by modulating the expression of the intestine specific homeobox (ISX), a retinoic acid-sensitive gene [57]. Therefore, we fed four-week-old *Bco1*^*−/−*^*/Ldlr*^*−/−*^ mice with a Standard-β-carotene diet for four weeks. After this, we switched the mice to a carotenoid-free diet without vitamin A or supplemented with 4 IU/g of vitamin A for four more days for fecal collection and tissue analyses. Mice fed the vitamin A diet exhibited upregulated *ISX* expression, a vitamin A-sensitive gene that blocks the expression of SR-BI [54], leading to decreased expression of SR-BI (Figure 7A, B). However, we did not observe differences in fecal β-carotene elimination between mice fed the two diets (Figure 7A, B).

## 4 Discussion

Over the past 20 years, research has led to the identification of carotenoid binding proteins and transporters previously recognized to interact with other lipids such as cholesterol [[15], [16], [17],^20^,^25^,^58^,^59^]. The contribution of LDLR in hepatic β-carotene uptake was first described by Quadro's group [26]. This report confirms and expands Quadro's findings by combining various experimental approaches. Our experiments also identify a novel carotenoid disposal pathway in mammals involving LDLR that operates independently of the carotenoid-cleaving enzymes.

To evaluate the effect of LDLR in mammals, we relied on *Ldlr*^*−/−*^ mice. These mice develop hyperlipidemia, which is exacerbated in the presence of dietary cholesterol (Figure 1C). This dramatic phenotype could be a confounding factor, which prompted us to perform our first study in *Bco1*^*−/−*^ and *Bco1*^*−/−*^*Ldlr*^*−/−*^ mice using both Standard (no cholesterol, 16% fat) and Western (0.3% cholesterol, 41% fat) diets (Figure 1A, Table 1). Under both experimental conditions, our results show that LDLR participates in the uptake of β-carotene in the liver and WAT, which account for the two largest carotenoid reservoirs in humans [[27], [28], [29]]. These results were supported by our LDLR over-expression studies, which revealed that hepatic LDLR not only participates in the uptake of β-carotene in *Bco1*^*−/−*^ mice, but also in the uptake of lutein in *Bco2*^*−/−*^ mice (Figure 3).

Visual observation and β-carotene quantifications in tissues initially suggested *Bco1*^*−/−*^*Ldlr*^*−/−*^ mice accumulated less β-carotene than *Bco1*^*−/−*^ mice (Figure 1). This observation raised an important question that, to our knowledge, was never formulated in the carotenoid field: Is it possible that carotenoids are eliminated independently of carotenoid-cleaving enzymes, and if so, is LDLR involved in this process? This gap in knowledge, together with accumulating evidence connecting carotenoid and cholesterol transport, prompted us to mine the literature to explore the role LDLR plays in cholesterol disposal. Fecal cholesterol disposal is derived from both biliary and non-biliary routes, in which LDLR plays a pivotal role in both. In hepatocytes, LDLR contributes to the elimination of cholesterol by providing cholesterol for bile acid synthesis. LDLR is also expressed in the basolateral membrane of the enterocyte, where it mediates TICE, which accounts for approximately 30% of total cholesterol elimination in humans [60]. LDLR over-expression in the intestine depletes circulating cholesterol to stimulate fecal cholesterol elimination in rodents [61], further supporting its role in TICE. This relatively unknown pathway prompted us to expand our perspective on LDLR's role in carotenoid disposal.

Our report provides compelling evidence for the contribution LDLR in the transintestinal elimination of carotenoids. First, *Bco1*^*−/−*^ and *Bco1*^*−/−*^*Ldlr*^*−/−*^ mice fed β-carotene for 12 weeks and then switched to a carotenoid-free diet showed that in the absence of LDLR, mice excrete less β-carotene (Figure 5A, B); second, β-carotene quantified in these mice at the level of three different portions of the intestine were greater in those mice expressing LDLR (Figure 5C, D); third, intestinal β-carotene positively correlated to LDLR expression in different intestinal regions of *Bco1*^*−/−*^ mice (Figure 5E); and forth, intraperitoneal injections of β-carotene resulted in the quick appearance of β-carotene in feces (Figure 6B).

Biliary excretion is a second pathway for carotenoid disposal that cannot be ignored. To date, only one study reported the presence of carotenoids in human bile [47]. The authors reported a positive correlation between biliary and plasma β-carotene levels, where biliary β-carotene was approximately 10-fold lower than the plasma concentration [47]. In 1992, Wang's group attempted to quantify biliary β-carotene disposal using ferrets, which are well-accepted experimental model to study carotenoid metabolism because they accumulate these compounds like humans do and express BCO1 and BCO2 [44]. Wang and colleagues canulated the bile duct and perfused radiolabeled β-carotene, however, the authors failed to detect either radiolabeled β-carotene or its cleavage products [62] in the bile. In the present study, we observed the presence of β-carotene in the bile under various experimental conditions (Figure 4, Figure 6E). Importantly, we observed a linear correlation between circulating and biliary β-carotene in *Bco1*^*−/−*^ mice, as it occurs in humans [47], providing further evidence that *Bco1*^*−/−*^ mice are an adequate model to study biliary carotenoid excretion (Figure 4F).

## 5 Limitations of the study

While our results cannot distinguish between the biliary and transintestinal routes, our data highlight the first evidence of a previously uncharacterized excretory pathway for carotenoids that likely involves a combination of the biliary and non-biliary routes. A constant flux of carotenoid disposal could operate until the elimination of all carotenoids in tissues, which is supported by comparable levels of fecal and intestinal β-carotene between four days and two weeks after the removal of dietary β-carotene (Figure 4, Figure 5A, B). Bile duct ligation experiments in mice lacking and expressing LDLR would be necessary to estimate the contribution of the biliary and non-biliary pathways, however, these surgical procedures can lead to confounding effects such as liver inflammation and cirrhosis [63,64]. Most of our studies were carried out in mice supplemented for several weeks using a single carotenoid. More physiologically relevant conditions using a mixture of carotenoids, or lower concentrations of these compounds, would be useful to confirm the contribution of LDLR in carotenoid elimination. Additionally, differences in the lipoprotein profile between *Bco1*^*−/−*^ and *Bco1*^*−/−*^*Ldlr*^*−/−*^ mice could contribute to differences in the excretion between the genotypes. Indeed, the distribution of β-carotene among lipoprotein species – (V)LDL and HDL – is dramatically different between genotypes (Figure 2B, E).

Clinical studies to confirm whether this novel pathway for carotenoid excretion is relevant to humans will be necessary. If active in humans, this pathway could deplete bodily carotenoid reserves when the consumption of carotenoid-rich diets is interrupted. It is possible that the continuous consumption of carotenoid-rich foods is required to sustain visual health and vitamin A homeostasis, as these compounds could be constantly depleted in our feces unnoticed. Lastly, we did not explore whether the fecal elimination of carotenoids other than β-carotene also occurs. However, our LDLR hepatic over-expression experiments showed comparable results in *Bco1*^*−/−*^ and *Bco2*^*−/−*^ mice fed β-carotene and lutein, respectively (Figure 3).

## CRediT authorship contribution statement

**Anthony P. Miller:** Writing – original draft, Visualization, Validation, Supervision, Project administration, Methodology, Investigation, Formal analysis, Conceptualization. **Walter C. Monroy:** Writing – review & editing, Resources, Methodology, Investigation, Formal analysis. **Gema Soria:** Writing – review & editing, Supervision, Resources, Methodology, Investigation. **Jaume Amengual:** Writing – review & editing, Writing – original draft, Visualization, Validation, Supervision, Software, Resources, Project administration, Methodology, Investigation, Funding acquisition, Formal analysis, Data curation, Conceptualization.

## Declaration of competing interest

The authors declare that they have no known competing financial interests or personal relationships that could have appeared to influence the work reported in this paper.

## Data Availability

Data will be made available on request.
