# Supplementary material for: The low-density lipoprotein receptor contributes to carotenoid homeostasis by regulating tissue uptake and fecal elimination
Source: Mol Metab. 2024 Aug 10;88:102007. doi: 10.1016/j.molmet.2024.102007 (PMC11382122; doi:10.1016/j.molmet.2024.102007)
Supplement: Multimedia component 1 [file mmc1.docx]

**Supplementary Figures**


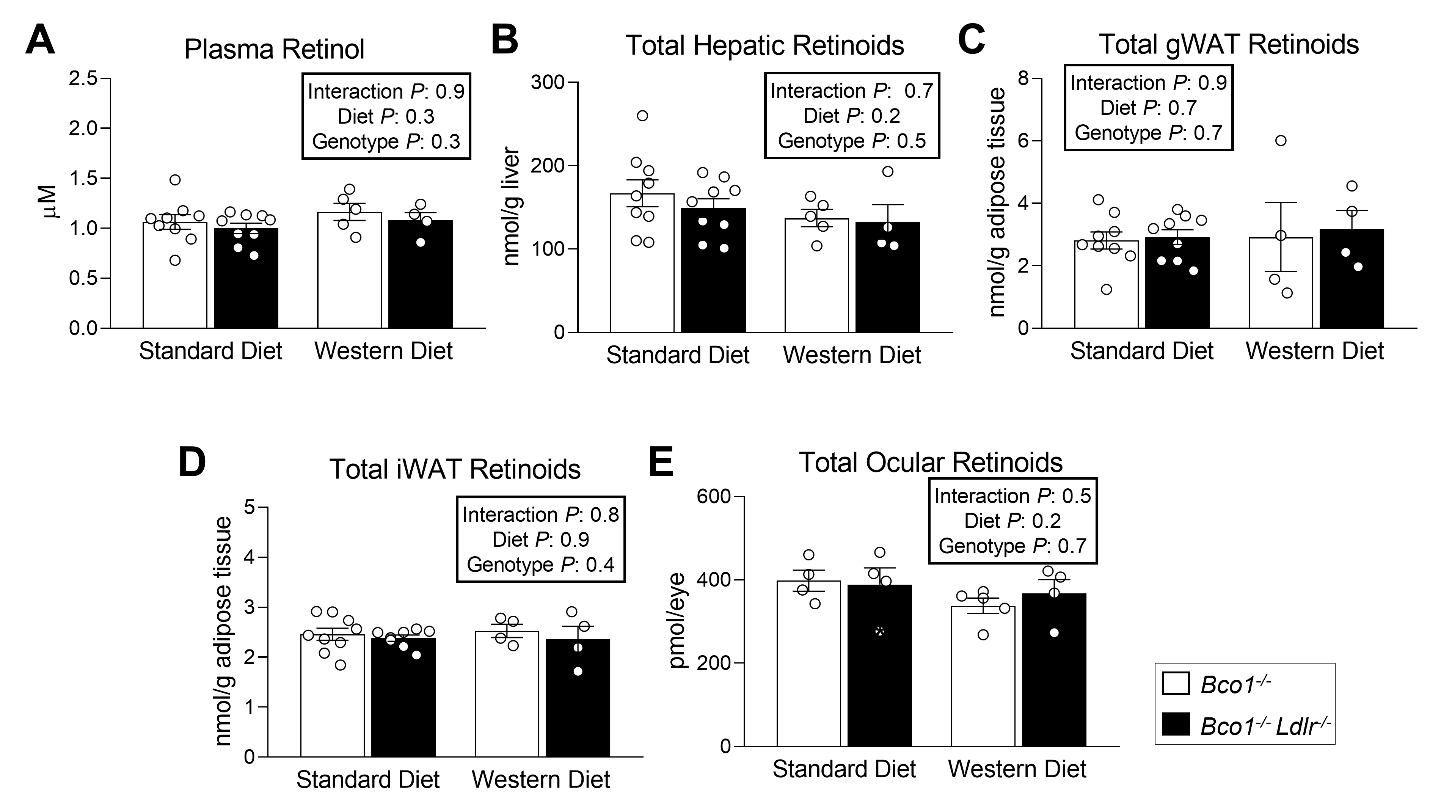


**Supplementary Figure 1.** Vitamin A homeostasis in *Bco1^-/-^* and *Bco1^-/-^/Ldlr^-/-^* mice fed Standard and Western diets supplemented with 50mg/kg of β–carotene for 12 weeks. **(A)** Plasma, **(B)** Hepatic, **(C)** Gonadal white adipose tissue (gWAT), **(D)** Inguinal white adipose tissue, and **(E)** Total ocular retinoid levels. N = 4 to 9 mice/group. Statistical differences were evaluated by two-way ANOVA. Overall p-values are displayed in the box.


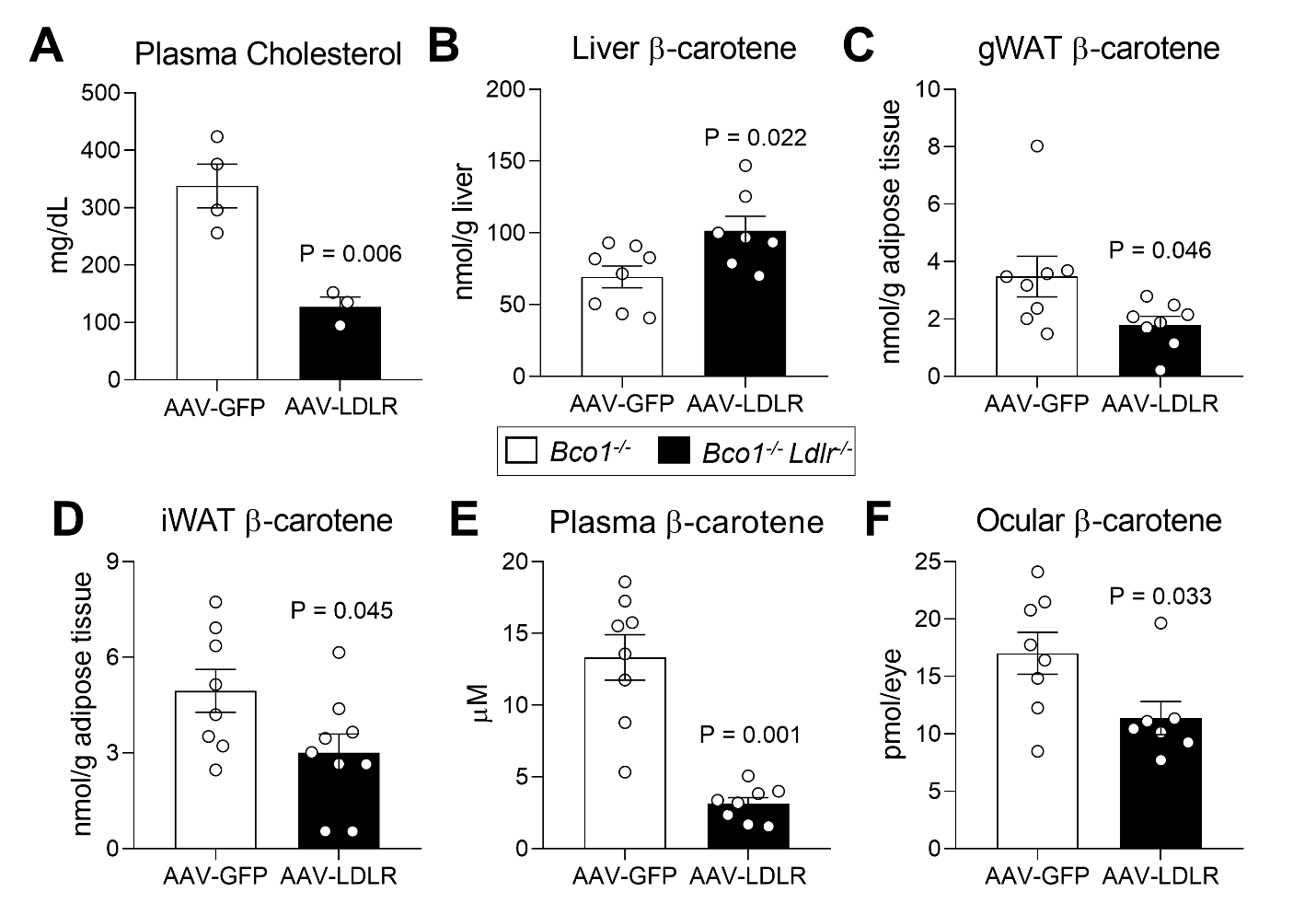


**Supplementary Figure 2.** Hepatic LDLR expression promotes carotenoid uptake in *Bco1^-/-^Ldlr^-/-^* mice. We injected *Bco1^-/-^Ldlr^-/-^* mice retro-orbitally with AAV-GFP or AAV-LDLR and immediately switched to Standard diet supplemented with 50mg/kg of β–carotene for four weeks. **(A)** Total plasma cholesterol levels *Bco1^-/-^ /Ldlr^-/-^* mice at 4 weeks. **(B)** Hepatic, **(C)** Gonadal white adipose tissue (gWAT), **(D)** Inguinal WAT (iWAT), **(E)** Plasma, and **(F)** Ocular β-carotene levels measured by HPLC. N = 3 to 8 mice/group Statistical differences were evaluated by two-tailed Student’s t-test. P-values are represented for each comparison. AAV, adeno-associated virus; GFP, green fluorescent protein.
